# 共价有机骨架材料在有毒有害物质萃取中的应用进展

**DOI:** 10.3724/SP.J.1123.2021.12004

**Published:** 2022-07-08

**Authors:** Wenmin ZHANG, Guancheng LIU, Wende MA, Min FANG, Lan ZHANG

**Affiliations:** 1.闽江师范高等专科学校, 福建 福州 350108; 1. Minjiang Teachers College, Fuzhou 350108, China; 2.福州大学, 食品安全与生物分析教育部重点实验室, 福建 福州 350116; 2. Ministry of Education Key Laboratory for Analytical Science of Food Safety and Biology, Fuzhou University, Fuzhou 350116, China

**Keywords:** 共价有机骨架材料, 农药, 持久性有机污染物, 塑化剂, 药物, 综述, covalent organic frameworks, pesticides, persistent organic pollutants, plasticizer, medicines, review

## Abstract

有毒有害物质的排放以及其可能具有的持久性和生物蓄积性,时刻危及人体健康甚至生命。因此,对环境、饮用水、食物和日用品中的有毒有害物质进行分析检测十分重要。对于复杂样品中痕量有毒有害物质的分析,样品预处理是一个至关重要的环节,直接影响分析方法的灵敏度和准确性。在有毒有害物质萃取中广泛应用的预处理技术包括固相萃取(SPE)、固相微萃取(SPME)、分散固相萃取(DSPE)、磁固相萃取(MSPE)等。在上述样品预处理技术中,吸附剂材料是最为核心的部分,它决定了预处理方法的选择性和效率。近年来,共价有机骨架(covalent organic frameworks, COFs)材料因其具有形貌结构多样、比表面积高、孔径可调、稳定性良好等优点,在样品预处理领域受到越来越多的关注。然而,COFs材料在萃取有毒有害物质方面的应用仍存在一些问题需要解决:(1)多数COFs是高度疏水的,这限制了它们在水基样品中的分散性,导致不良的萃取效果;(2)COFs材料主要依靠*π-π*堆积等相互作用对疏水性目标物进行高效萃取,但不利于极性有毒有害物质的萃取;(3)多数COFs材料存在合成工艺复杂、生产成本高、量产困难等问题。该文对近几年COFs材料在有毒有害物质萃取过程中的研究进展进行了总结和评述。最后,展望了COFs材料在该领域中的应用前景,为进一步研究基于COFs材料的预处理技术提供了参考。

## Abstract (en)

Toxic and hazardous substances constitute a category of compounds that are potentially hazardous to humans, other organisms, and the environment. These substances include pesticides (benzoylureas, pyrethroids, neonicotinoids), persistent organic pollutants (polycyclic aromatic hydrocarbons, polychlorinated biphenyls, perfluorinated compounds), plasticizers (phthalate esters, phenolic endocrine disruptors), medicines (sulfonamides, non-steroid anti-inflammatory drugs, tetracyclines, fluoroquinone antibiotics), heterocyclic aromatic amines, algal toxins, and radioactive substances. Discharge of these toxic and harmful substances, as well as their possible persistence and bioaccumulation, pose a major risk to human health, often to the extent of being life-threatening. Therefore, it is important to analyze and detect toxic and hazardous substances in the environment, drinking water, food, and daily commodities. Sample pretreatment is an imperative step in most of the currently used analytical methods, especially in the analysis of trace toxic and harmful substances in complex samples. An efficient and fast sample pretreatment technology not only helps improve the sensitivity, selectivity, reproducibility, and accuracy of analytical methods, but also avoids contamination of the analytical instruments and even damages the performance and working life of instruments. Sample pretreatment techniques widely used in the extraction of toxic and hazardous substances include solid-phase extraction (SPE), solid-phase microextraction (SPME), and dispersed solid-phase extraction (DSPE). The adsorbent material plays a key role in these pretreatment techniques, thereby determining their selectivity and efficiency. In recent years, covalent organic frameworks (COFs) have attracted increasing attention in sample pretreatment. COFs represent an exciting new class of porous crystalline materials constructed via the strong covalent bonding of organic building units through a reversible condensation reaction. COFs present four advantages: (1) precise control over structure type and pore size by consideration of the target molecular structure based on the connectivity and shape of the building units; (2) post-synthetic modification for chemical optimization of the pore interior toward optimized interaction with the target; (3) straightforward scalable synthesis; (4) feasible formation of composites with magnetic nanoparticles, carbon nanotubes, graphene, silica, etc., which is beneficial to enhance the performance of COFs and meet the requirement of diverse pretreatment technologies. Because of the well-defined crystalline porous structures and tailored functionalities, COFs have excellent potential for use in target extraction. However, some issues need to be addressed for the application of COFs in the extraction of toxic and hazardous substances. (1) For the sample matrix, most of the reported COFs are highly hydrophobic, which limits their dispersibility in water-based samples, leading to poor extraction performance. COFs with good dispersibility in water-based samples are urgently required. (2) Besides, COFs rely on hydrophobic interaction, size repulsion, *π-π* stacking, and Van der Waals forces to extract target substances, but they are not effective for some polar targets. Thus, it is necessary to develop COFs with high affinity for polar toxic and hazardous substances. (3) Methods for the synthesis of COFs have evolved from solvothermal methods to room-temperature methods, mechanical grinding, microwave-assisted synthesis, ion thermal methods, etc. Most of the existing methods are time-consuming, laborious, and environmentally unfriendly. The starting materials are too expensive to prepare COFs in large quantities. More effort is required to improve the synthesis efficiency and overcome the obstacles in the application of COFs for extraction. This article summarizes and reviews the research progress in COFs toward the extraction of toxic and hazardous substances in recent years. Finally, the application prospects of COFs in this field are summarized, which serves as a reference for further research into pretreatment technologies based on COFs.

有毒有害物质是指会对人、其他生物或环境带来潜在危害特性的物质。有毒有害物质的大量使用和排放以及其可能具有的持久性和生物蓄积性,使得人类容易在生产、生活中接触到,从而危及人类健康甚至生命^[1⇓-3]^。因此,对环境、饮用水、食物和日用品中的有毒有害物质进行分析检测十分重要。

样品预处理是几乎所有分析方法的基础和必要步骤,特别是对复杂基质样品中痕量目标物的分析^[4]^。高效、快速的样品预处理技术不仅有利于提高分析方法的检测灵敏度、选择性、重现性和准确性,而且能避免污染仪器乃至损害仪器性能以及寿命^[5]^。样品预处理的萃取方式可分为溶剂型和吸附型,而吸附型萃取由于其有效性、便捷性而得到广泛应用。吸附型萃取包括固相萃取(solid-phase extraction, SPE)、固相微萃取(solid-phase microextraction, SPME)、磁性固相萃取(magnetic solid-phase extraction, MSPE)、分散固相萃取(dispersed solid-phase extraction, DSPE)等。吸附剂材料是吸附型萃取技术中最重要的组成部分,它决定了吸附萃取方法的选择性和效率^[6⇓⇓-9]^。目前,许多种类的多孔材料被用于吸附型萃取当中,包括碳基材料^[10,11]^、金属有机骨架(metal organic frameworks, MOFs)^[12,13]^、金属氧化物^[14]^、分子印迹聚合物(molecular imprinted polymer, MIP)^[15,16]^、大分子聚合物^[17]^以及共价有机骨架(covalent organic frameworks, COFs)等。

其中,COFs是由有机单体分子通过可逆的缩合反应形成的一类新型多孔晶体材料^[18,19]^,在气体储存^[20]^、催化^[21,22]^、传感器^[23]^、光电子学^[24]^等方面得到广泛研究。形成COFs的单体种类的多样性决定了COFs种类的多样性。组成COFs的基团主要有硼氧六环、硼酸酯、三嗪基、亚胺以及基于亚胺键的其他基团等,因此将COFs材料大致分成3大类:含硼类、亚胺类、三嗪类^[25⇓-27]^。COFs的多孔结构及高比表面积能够为吸附目标物提供足够多的吸附位点以及高的吸附容量。不仅如此,COFs还具有可修饰功能化的特点,多样化的官能团使得其能通过多种作用力吸附目标物,包括疏水作用、氢键作用、静电相互作用、*π-π*堆叠作用、配位作用、主客体作用等。COFs还具有密度低、通道结构可调、骨架结构稳定等优点^[28]^。COFs的上述特点对于吸附目标物有着至关重要的作用,研究者能够根据实际需求选择合适的COFs材料在不同基质下对多种分析目标物进行萃取。与此同时,COFs的合成方式由最初耗时长、反应条件苛刻的溶剂热法^[25,29⇓-31]^,逐渐发展为室温合成法^[32,33]^、机械合成法^[34,35]^、微波辅助合成法^[36,37]^、离子热法^[38]^等,通过优化合成条件,提高合成效率,减少COFs材料在样品预处理应用中的阻碍。此外,COFs能通过与磁性纳米粒子、碳纳米管、石墨烯、二氧化硅等其他种类材料形成复合材料,有利于拓展COFs材料的性能,满足不同应用场景下的不同需求。本文对COFs材料及其复合材料在有毒有害物质萃取过程中的应用进展进行总结和评述,并对其发展前景进行了展望。

## 1 农药

农药的使用有力地推动了农业生产的发展,但是大量的农药污染事件也引起人们对农药安全限量的关注。许多农药在食物、环境中的高水平残留以及累积,对人们的生命健康构成严重威胁,因此对食物、环境甚至人体中的农药进行检测显得至关重要。COFs材料具有的大共轭结构能与绝大多数农药分子形成*π-π*堆叠作用,同时二者之间亦可能存在氢键作用、静电作用、疏水作用以及范德华力等其他作用力;分子尺寸较小的农药分子亦能轻松进入COFs的孔道结构,上述原因使得COFs对农药具有优异的富集作用。

### 1.1 苯甲酰脲类杀虫剂

苯甲酰脲类杀虫剂(BUs)是一类功能强大的昆虫生长调节剂,具有生物活性高、环境持久性差、易降解等优点,在农业上得到了广泛的应用。然而,它们在农产品中的残留可能导致慢性暴露和长期毒性作用^[39,40]^。Wang课题组^[41]^通过采用快速、环保、易操作的研磨方法,以1,3,5-三甲基苯间苯三酚(Tp)与4,4-偶氮二苯胺(Azo)为单体,制备了Tp-Azo作为SPE墨盒吸附剂用于萃取果汁、土豆、白萝卜中的BUs,结合高效液相色谱法(HPLC)进行测定,其方法获得良好的线性(相关系数*R*≥0.9956)、较低的检出限(0.05~0.10 ng/mL, *S/N*=3)、较高的回收率(68.7%~92.3%)。随后,他们课题组^[42]^再次通过研磨的方法,以2,6-二氨基蒽醌(DAAQ)和1,3,5-三甲酰间苯三酚(TFP)为基本单体,制备了DAAQ-TFP作为SPE墨盒吸附剂,并将其用于萃取环境水样、果汁、水果和蔬菜中的BUs。由于具有大的表面积、高的孔隙率、良好的热化学稳定性、丰富的羟基和氨基,DAAQ-TFP表现出对BUs较高的吸附能力。该方法进一步降低了检出限(0.02~0.06 ng/mL, *S/N*=3),提高了回收率(85.5%~112.7%)。此外,Wang课题组^[41,42]^详细探究了DAAQ-TFP和Tp-Azo对BUs的吸附机理,结果均证明它们与BUs之间的*π-π*堆叠作用以及氢键作用对吸附行为起着至关重要的作用。

### 1.2 拟除虫菊酯类杀虫剂

拟除虫菊酯类杀虫剂(PYs)是环境卫生和农作物病虫害防治方面最重要的一类杀虫剂,具有优异的生物活性和良好的环境相容性,并且对哺乳动物的毒性一般较低。尽管如此,PYs对于蜜蜂、水生动物的危害以及所带来的环境风险也逐渐引起了人们重视^[43,44]^。Wu等^[45]^制备了一种由Tp和对苯二甲酸二肼(TPDH)有机单体构成的Tp-TPDH,并将其包覆在聚多巴胺修饰的不锈钢纤维上。该萃取纤维具有良好的重现性和热稳定性,与目标物之间有*π-π*堆叠作用、氢键作用、疏水作用以及范德华力,表现出对目标物的高吸附性能。随后,将其用于顶空固相微萃取,结合气相色谱-电子捕获检测器(GC-ECD),检测苹果和黄瓜样品中的PYs。该研究所建立的方法具有线性范围广(0.11~0.23 μg/kg)、检出限(0.11~0.23 μg/kg)和定量限(0.37~0.77 μg/kg)低、快速和简便等优点。此外,利用分子印迹技术有利于降低检测方法的检出限及定量限。Ji等^[46]^在室温下以苯戊酸盐为模板,在催化剂的作用下,1,3,5-三(4-氨基苯基)苯(TAPB)和Tp快速成功制备了亚胺类分子印迹TAPB-Tp,用作SPE墨盒吸附剂。亚胺类分子印迹TAPB-Tp表现出对4个结构相似含氰基的拟除虫菊酯类杀虫剂的高选择性,结合HPLC测定水果、蔬菜和传统中药中拟除虫菊酯。建立的方法简便,选择性好,具有低的检出限(0.011~0.018 ng/g)、定量限(0.036~0.060 ng/g)和较高的回收率(>95%),为食品中PYs的测定提供了一种新方法。

### 1.3 其他种类农药

COFs还被用于萃取其他种类的农药。Ji等^[47]^以4-氨基苯硫醚为原料,通过巯基-烯键反应合成了氨基修饰的共价有机骨架NH_2_@COFs材料。氨基的引入有助于提高吸附剂与羧酸类农药中阴离子基团的相互作用,并且赋予了吸附剂良好的亲水性,使NH_2_@COFs在水相介质中能更充分地与目标物接触。探究其吸附机理,证实NH_2_@COFs对羧酸类农药的吸附为单层吸附,进一步验证NH_2_@COFs的氨基与羧酸类农药中羧基的相互作用为主要作用力。建立的NH_2_@COFs-SPE-HPLC方法线性范围宽(0.2~100 ng/mL, *R*>0.999)、检出限(LOD)低(0.04~0.20 ng/mL)、精密度好(RSD≤8.7%, *n*=6),成功地分析了6种环境水样中的羧酸类农药。Lu等^[48]^合成了硝基功能化的核壳结构的磁性共价有机骨架Fe_3_O_4_@COFs-(NO_2_)_2_,对6种新烟碱类杀虫剂残留进行了MSPE处理,并结合HPLC测定蔬菜样品中的新烟碱类杀虫剂。除了Fe_3_O_4_@COFs-(NO_2_)_2_与新烟碱类之间的*π-π*堆叠作用、氢键作用,COFs孔道上的-NO_2_还提供了额外的强亲水相互作用。相较于其他已经报道^[49⇓-51]^的方法,该研究所建立的方法提取时间短(10 min),线性范围宽(0.1~30 ng/mL), LOD低(0.02~0.05 ng/mL, *S/N*=3),富集因子大(170~250),回收率高(77.5%~110.2%)。

## 2 持久性有机污染物

持久性有机污染物(POPs)是一类具有持久性、生物积累性、易于迁移性的并对人类及其他生物具有毒副作用的有机污染物,已成为全球关注的热点^[52⇓-54]^。它包括多环芳烃(PAHs)、多氯联苯(PCBs)、全氟化合物(PFCs)等。

### 2.1 多环芳烃

PAHs主要来源于有机物的不完全燃烧,可通过呼吸、皮肤接触、个人习惯(如吸烟和饮食摄入)等途径被人体吸收并转化。PAHs及其衍生物具有潜在的致癌和致突变作用,在体内和体外均可能对人体健康有害^[55,56]^。基于COFs结构中大共轭骨架与PAHs之间存在的*π-π*堆叠作用,目前已有多种COFs通过不同的萃取方式用于PAHs的分析检测中^[57⇓⇓⇓⇓-62]^。Tian等^[62]^在温和可控的条件下,将四(4-氨基苯基)卟啉(TAPP)和三(4-甲酰基苯基)胺(TFPA)组成的新型材料TAPP-TFPA,作为微萃取的涂层逐层固定在不锈钢纤维上。将纤维应用于顶空固相微萃取法萃取水溶液中的PAHs,之后用火焰电离检测器进行气相色谱分析。该研究所建立的方法具有线性范围宽(0.1~50 μg/L)、检出限低(0.006~0.024 μg/L, *S/N*=3)、重现性好(RSD≤9.98%, *n*=3)的优点,说明了新开发涂层的优异适用性。

快速分析是分析化学的一个发展趋势。Zhang等^[59]^利用TAPB和对苯二甲醛(TPA)单体合成磁性共价有机骨架Fe_3_O_4_@TAPB-TPA,将其作为吸附剂和基底,通过不同模式的表面辅助激光解吸/电离飞行-质谱分析(SALDI-TOF-MS)测定PM_2.5_中的PAHs及其衍生物。由于Fe_3_O_4_@TAPB-TPA具有高富集能力和无基质干扰等优点,该方法相较于传统气相色谱-质谱联用仪(GC-MS)和液相色谱-质谱联用仪(LC-MS),对PM_2.5_中多环芳烃及其衍生物测定时,样品制备更加简单,仪器运行时间更短,分析速度更快。

### 2.2 多氯联苯

PCBs是一类持久性脂溶性有机污染物,广泛存在于环境和食品中,并且可以在生物体内进行衍生化^[63,64]^。Guo等^[65]^在室温条件下,将由1,3,5-三(对甲酰基苯基)苯(TFPB)和联苯胺(BD)组成的TFPB-BD通过原位生长的方式键合到氨基化不锈钢纤维上,用于固相微萃取PCBs。采用研制的TFPB-BD键合纤维,建立了水产品中痕量PCBs的GC-MS/MS分析新方法。制备的新型TFPB-BD键合纤维具有较高的热稳定性和化学稳定性,并具有良好的重复使用性能。将其应用于鲶鱼、黑鱼、基围虾、欧洲鲫鱼、鲷鱼、对虾等水产品中多氯联苯的萃取,结合GC-MS/MS,方法具有高富集因子(4471~7488)、低检出限(0.07~0.35 ng/L)、良好的重现性(RSD≤9.8%, *n*=3)和准确性(RSD≤8.8%, *n*=6)。适配体是一种短单链DNA/RNA分子,和靶点之间的相互作用包括范德华力、氢键作用和电子受体和供体相互作用,类似于免疫学中对抗体和抗原的识别^[66]^。Jiang等^[67]^制备了一种基于适配体功能化的磁性COFs吸附剂,该吸附剂可选择性提取痕量的羟基化多氯联苯(OH-PCBs)。该材料兼具磁芯的超顺磁性、COFs的高表面积和多孔结构、适配体的高比亲和力等优点。结合高效液相色谱-质谱联用技术,利用COFs与OH-PCBs之间的*π-π*堆叠作用、氢键作用及适配体与目标物的高亲和力,该适配体功能化磁性COFs成功在人血清中捕获OH-PCBs。该方法的线性范围为0.01~40 ng/mL,相关系数较好(*R*=0.9986),检出限相比于绝大多数非分子印迹技术都低(2.1 pg/mL)。该材料具有良好的重复使用性能,可重复利用至少10次,回收率在90%以上。

### 2.3 全氟化合物

PFCs是一种新型的有机污染物,广泛应用于染料、纺织品、食品包装、润滑剂等领域,研究表明其对肝脏、心血管系统和神经调节等有不良影响。目前已经报道多种COFs材料或复合材料被用于萃取PFCs^[68⇓⇓⇓⇓-73]^。传统COFs可通过疏水作用、静电作用、氢键作用等有效吸附PFCs,选择氟原子取代的COFs单体制备氟原子功能化的COFs,则能够利用氟氟亲和力对PFCs进行选择性吸附。Ji等^[71]^采用微波辅助合成法,制备了一种由2,3,5,6-四氟-4-吡啶腈(TFPC)与2,3,6,7,10,11-六羟基三苯(HHTP)反应生成的TFPC-HHTP,并将TFPC-HHTP作为SPME纤维涂层用于全氟烷基化合物(PFASs)的快速萃取。利用SPME纤维结合超高效液相色谱-串联质谱仪(UPLC-MS/MS)对实际水样中的痕量PFASs进行检测分析。在最佳微萃取条件下,TFPC-HHTP-SPME-UPLC-MS/MS方法线性范围宽(0.01~1000 ng/L), LOD低(0.0020~0.0045 ng/L),重复性好(RSD≤7.9%, *n*=6)。实验结果表明,TFPC-HHTP不仅具有较大的比表面积,能有效增加对目标物的吸附位点,而且TFPC-HHTP中的N原子与PFASs容易形成分子间氢键,极大地提高了对目标物的萃取性能。Zhang等^[73]^通过原位生长策略成功制备氟化磁性共价有机骨架(Fe_3_O_4_@TpPa-F_4_),并作为一种新型的氟磁性固相吸附剂用于富集PFCs。制备的Fe_3_O_4_@TpPa-F_4_具有较好的孔隙率、较大的表面积、较高的氟含量、较强的磁响应性、良好的化学稳定性和热稳定性。基于COFs层独特的性能以及强力的氟氟作用,Fe_3_O_4_@TpPa-F_4_表现出对PFASs较高的吸附能力和选择性。通过将其与HPLC-MS/MS相结合,建立了一种对超痕量PFCs的高灵敏检测方法。该方法具有线性范围宽(0.1~250 ng/L)、检出限极低(0.005~0.05 ng/L, *S/N*≥3)、前处理时间短(30 min)、富集因子高(22~101)、重现性好等优点。实际样测试结果表明,该方法适用于不同品牌和包装的牛奶样品中PFCs的分析检测。

## 3 塑化剂

### 3.1 邻苯二甲酸酯类

邻苯二甲酸酯类化合物(PAEs)作为增塑剂广泛应用于各种塑料制品中,以提高塑料制品的柔韧性和性能^[74]^。PAEs广泛应用于塑料包装、塑料容器和化妆品中,目前已成为人们日常接触的最常见的芳香族化学物质^[75]^。然而PAEs具有致癌性,能干扰内分泌系统紊乱和阻碍生殖器官发育^[76]^。大的共轭骨架使得COFs对于PAEs的萃取效果十分出色^[77⇓⇓⇓-81]^。Yan等^[78]^通过简便高效的方法制备亲水Fe_3_O_4_@PDA@TbBd纳米球。新合成的纳米球具有一个独特的*π-π*电子系统、良好的亲水性和较强的磁响应、常规的多孔结构和高表面积,对PAEs表现出优异的萃取性能。随后,将其与GC-MS相结合,建立了一种复杂基质中PAEs的检测方法,该方法具有较低的检出限(0.0025~0.01 ng/mL)、较宽的线性范围(50~8000 ng/mL)和较高的回收率(92.3%~98.9%)。在实际应用中,在基质非常复杂的人血浆样品中检测到9种PAEs,得到的效果令人满意。此外,简单快速的磁化方法有利于推广COFs的应用。Pang等^[80]^通过一种简单、快速的共沉淀法合成磁性共价有机骨架吸附剂。先合成出TpBD,之后将其研磨成纳米片,最后通过共沉淀法制得磁性材料TpBD@Fe_3_O_4_。将制备的TpBD@Fe_3_O_4_用于饮料样品中高达15种PAEs的磁性固相萃取过程,结合GC-MS/MS进行测定。由于PAEs与TpBD之间的疏水作用和孔径选择性,该方法具有良好的线性度(*R*≥0.9956)、较低的检出限(0.005~2.748 ng/mL)和较高的准确度,极大地降低了基质的影响,成功应用于8种饮料中15种PAEs的测定,回收率高(79.3%~121.8%),可靠性强(RSD≤11.9%, *n*=3)。

### 3.2 酚类内分泌干扰物

酚类内分泌干扰物(PEDs),包括双酚A、双酚F、壬基酚和辛基酚等物质,作为增塑剂被广泛使用。然而PEDs能够模拟内源性激素作用,从而干扰内分泌器官功能,对人类健康造成包括生殖功能障碍、出生缺陷、代谢紊乱和某些恶性肿瘤在内的严重威胁^[82⇓-84]^。因为COFs的多孔结构、大的比表面积以及能与PEDs通过*π-π*堆叠及氢键相互作用,所以有多种COFs被应用于萃取分离PEDs^[74,85⇓⇓⇓-89]^。Liu等^[87]^以典型的大表面积、化学稳定性好的球形TpBD为吸附剂,封装于SPE墨盒中,对PEDs进行预富集,结合HPLC进行分析。TpBD优异的化学稳定性和分散性保证了墨盒能满足20次以上的吸附洗脱循环和可接受的回收率(>80%)。该方法快速、可靠、灵敏度高(LOD=0.056~1.123 μg/L)。Deng等^[88]^在室温条件下以TAPB和TPA为单体制备了具有核壳结构的磁性共价有机骨架Fe_3_O_4_@TAPB-TPA,作为吸附剂对PEDs进行磁性固相萃取。该吸附剂具有大的比表面积和丰富的-C=N-、-COOH、-NH_2_等基团,可与目标分子形成*π-π*堆叠、氢键和其他作用力。采用HPLC测定酚类化合物的含量。该方法具有检出限低(0.08~0.21 ng/mL)、线性范围宽(0.5~1000 ng/mL)、重复性好(RSD≤5.21%, *n*=5)等优点。将所建立的方法应用于实际饮料样品中4种目标污染物的测定,可获得不错的回收率(81.3%~118.2%)。

## 4 药物污染物

药物被广泛应用于养殖业以及医疗领域,过量使用使得它们被排放到环境中,有些则残留于食物当中,对人生命健康构成威胁。

### 4.1 磺胺类药物

磺胺类药物(SAs)是一类含有磺胺化学结构的合成抗生素,广泛应用于动物饲料中,能够预防动物疾病,提高饲料转化率,促进动物生长发育^[90]^。然而,食品中SAs残留的存在可能会产生一些负面影响,如细菌耐药性的产生和人体致癌风险的提高^[91]^。SAs极性较强并且具有苯环基团,能与COFs形成氢键以及*π-π*作用^[92⇓⇓⇓-96]^。Xu等^[95]^将多孔共价有机氮骨架(PCONFs)作为固相萃取盒的填料,快速从复杂样品中提取8种磺胺类抗生素,之后通过LC-MS/MS在多反应监测(MRM)模式下进行检测。PCONFs对SAs具有优异的萃取性能,原因包括:(1)PCONFs和目标物SAs均含有电负性原子N,能形成氢键相互作用;(2)SAs中的苯环和PCONFs之间的*π-π*堆叠作用,有利于提高PCONFs对SAs的亲和力;(3)PCONFs的高表面积有利于与SAs接触;(4)SAs的分子大小小于PCNOFs的平均孔径,因此SAs可以很容易地接触到PCONFs的内部吸附位点,从而提高对SAs的吸附量。基于PCONFs的SPE与LC-MS/MS相结合检测SAs的方法,具有较宽的线性范围(2.5~1000 ng/L)、较低的LOD(0.14~2.0 ng/L)、较好的重复性(RSD≤12.9%, *n*=6)、较短检测时间(5.5 min)等优点。将该方法成功应用于实际水、牛奶和鸡肉样品中8种SAs的分析。目前COFs材料大多是疏水性的,对于极性较强的SAs选择性不高。Yang等^[96]^将*β*-环糊精(*β*-CD)修饰在磁性TpBD上,获得Fe_3_O_4_@TpBD@*β*-CD以增强极性,更有利于吸附SAs。Fe_3_O_4_@TpBD@*β*-CD萃取SAs效果显著,主要原因包括*β*-环糊精的超分子作用以及富集能力、COFs的多孔结构、Fe_3_O_4_@TpBD@*β*-CD与SAs之间的氢键作用、疏水作用、*π*电子堆叠。将所制备的材料用作磁性固相萃取吸附剂,结合HPLC对SAs进行富集检测,方法具有良好的线性(*R*≥0.9968)和低的检出限(0.8~1.6 μg/kg)。之后成功地应用于对鸡肉、猪肉和牛肉等实际样品中9种SAs的检测,效果优异。

### 4.2 非甾体类抗炎药

非甾体类抗炎药(NSAIDs)是一类具有解热、抗炎、镇痛作用的化合物,用于治疗风湿病、炎症和疼痛等。NSAIDs主要通过人体尿液、废水和过期药物的丢弃,排放到生态环境中。NSAIDs很难被生物降解,使其容易在环境中积累,从而对人类和其他生物构成威胁^[97⇓-99]^。Zhu课题组^[100]^通过水热法构建了一种新型共价有机骨架功能化聚(苯乙烯-二乙烯基苯-甲基丙烯酸缩水甘油酯)复合材料(COFs@PS-GMA)。COFs@PS-GMA具有多孔结构、高的比表面积和良好的化学稳定性。COFs@PS-GMA与非甾类抗炎药具有*π-π*堆积相互作用,其能快速萃取废水样品中的非甾体抗炎药。将COFs@PS-GMA作为注射器内SPE吸附剂,结合HPLC,用于测定环境水样中的7种非甾体抗炎药,该方法具有良好的线性(0.005~5.0 μg/mL)以及低的定量限(0.54~2.74 μg/L)。之后,他们课题组^[101]^采用非均相成核生长的方法制备了一种由无定形种子和分子印迹共价有机骨架壳组成的新型纳米复合材料。先将TPB、BD反应包覆在氨基化硅球上,将其作为无定型种子,之后以布洛芬为假模板,在无定型种子上生长分子印迹(MI)共价有机骨架材料,去除模板得到MI-COF@SiO_2_复合材料。该复合材料对非甾类抗炎药具有较高的吸附能力,这主要来源于其较大的比表面积以及材料与目标物之间的*π-π*堆积相互作用。将MI-COF@SiO_2_复合材料作为固相萃取吸附剂,结合HPLC,建立测定非甾类抗炎药的方法。该方法有良好的线性范围(0.002~1.0 μg/mL)以及可接受的可重复性,并且进一步降低了检出限(0.38~2.92 μg/L)。该方法成功地应用于环境水样品中非甾体抗炎药的分析,回收率良好(77%~122%)。

### 4.3 其他种类药物污染物

Wang等^[102]^采用简便的静电纺丝方法制备了一种新型共价有机骨架SCU1复合纳米纤维(PAN@SCU1),并将其作为移液管尖端固相萃取(PT-SPE)方法的吸附剂,用于食品中3种四环素(TCs)的萃取。制备的PAN@SCU1纳米纤维兼具电纺纳米纤维和SCU1的特性,可提高电纺纳米纤维的吸附能力,避免纳米尺寸的COFs直接作为吸附剂在PT-SPE中引起泄漏和高压的问题。PAN@SCU1纳米纤维表现出对TCs的高萃取效率,原因基于多个机制,如SCU1纳米纤维之间的静电作用、氢键作用、疏水作用和*π-π*堆积作用。将PT-SPE与HPLC相结合,建立了一种食品中TCs的高灵敏检测方法。该方法具有检出限(0.6~3 ng/mL)和定量限(2~10 ng/mL)低、日内和日间精密度好等优点。该方法首次成功用于草鱼和鸭肉样品中TCs残留量的测定。不仅如此,Wen等^[103]^和Wang等^[104]^分别制备不同的磁性COFs用于萃取检测氟诺喹酮抗生素。Li等^[105]^则制备磁性COFs用于吸附三氯生和三氯卡班。以上结果均表明,COFs材料在萃取检测食品或环境中药物污染物的良好应用。

## 5 其他有毒有害物质

杂环芳香族胺(HAAs)是氨基酸、肌酸、肌酸酐和碳水化合物的热解产物,已被认为是强有力的诱变和致癌物质^[106,107]^。Zhang等^[108]^制备新型双壳层TpBD磁性纳米球吸附材料,以实现对14种杂环芳香族胺(HAAs)的简单、快速、高选择性捕获。该吸附材料具有优异的分散性能、高的稳定性、优异的重复使用性能。通过量子化学理论计算,直接地量化描述TpBD和杂环芳香胺之间的多种相互作用力,包括*π-π*作用、氢键作用、阳离子-*π*作用、静电作用及离子交换,证实TpBD对HAA的杰出吸附能力。结合UPLC-MS/MS萃取检测14种HAAs,其方法回收率高(95.4%~129.3%),检出限(0.14~0.46 pg/mL, *S/N*=3)和定量限(0.41~1.37 pg/mL, *S/N*=10)均明显优于已报道的CE-MS和HPLC-MS的工作^[109,110]^。在实际样测试中,该方法成功地检测出了吸烟者尿液样本中的14种HAAs。通过与其他种类材料复合以改善硼酸酯类COFs的水稳定性,是拓展硼酸酯类COFs应用的行之有效的方法。Liang等^[111]^则利用光化学合成法将黄瓜状COFs(CTC)包覆在磁性碳纳米管(MCNT)上制得CTC@MCNT,以改善CTC的水稳定性。将其作为磁性固相萃取吸附剂,富集复杂样品炸鸡和烤牛肉中的9种HAAs,结合UPLC-MS/MS进行测定。基于主客体作用及静电相互作用,CTC@MCNT对HAAs具有良好的吸附性能。该研究所建立的方法表现出宽的线性范围(0.05~50 ng/g),高的灵敏度(LOD=0.0058~0.025 ng/g)。此外,实际样测试结果表明,该方法具有良好的回收率(73.0%~117%)、分析结果可靠(RSD≤9.1%, *n*=3)等优点。

藻毒素是由藻类产生的,可在海产品体内富集,人类食用藻毒素污染的海产品会危及人体健康^[112]^。Romero等^[113]^则将合成出的磁性COFs-(mTpBD-dimethyl)应用于冈田海绵酸(OA)和合鳍藻毒素(DTX-1)等海洋毒素的富集。得益于良好的孔隙结构及高的比表面积,mTpBD-dimethyl对OA和DTX-1的最大吸附容量分别高达812和830 mg/g,比常用的非磁性大孔树脂分别增大了500倍及300倍。以2-丙醇为洗脱剂,两种生物毒素的解吸效率几乎达到定量,经过连续5个吸附/解吸循环,复合材料的吸附能力损失较小,可回收利用。此外,吸附循环后结晶度的保留也突出了复合材料在海水中的稳定性。

由于核电广泛使用、大规模铀矿开采、核事故、核废料处置不当等原因,大量放射性铀以U $O_{2}^{2+}$ 的形式渗入环境中,引起人们在健康和环境方面的担忧^[114]^。目前,基于Schiff碱反应的各种COFs易受辐射、酸、碱的影响,极大地限制了在萃取放射性核素中实际应用以及再生。Cui等^[115]^报道了一种*sp*^2^碳共轭荧光共价有机骨架材料(TFPT-BTAN-AO),碳碳双键的连接使它具有良好的化学、热和辐射稳定性。对U $O_{2}^{2+}$ 的吸附能力高达427 mg/g,这是由于TFPT-BTAN-AO具有丰富的选择性铀结合基团。通过一系列表征手段证明,TFPT-BTAN-AO上的氨基与羟基均能与U $O_{2}^{2+}$ 存在配位作用,吸附过程是一个化学过程。此外,它具有超快的响应时间(2 s)和超低的检出限(6.7 nmol/L),适用于U $O_{2}^{2+}$ 的现场和实时监测,并且使用后的TFPT-BTAN-AO能通过碳酸钠溶液的清洗,重复使用。

## 6 结论与展望

综上所述,COFs由于其能通过多种相互作用吸附目标物,同时具有密度低、比表面积大、孔隙结构突出、通道结构可调、骨架结构稳定等优点,在有毒有害物质和生物分析的样品前处理领域成为一种优异的吸附剂材料。但是,仍旧存在一些问题影响COFs材料在样品前处理领域中的应用。目前的COFs合成方法费时、费力、环境污染大并且单体价格昂贵难以大量制备,不利于COFs材料的应用推广。因而,需要通过优化合成方法,开发出制备简便、选择性好、绿色环保、成本低的COFs,以便其在样品前处理领域得到更好的应用。此外,大多数报道的COFs是高度疏水的,限制了其在吸附亲水性化合物及在水溶剂中的应用。并且COFs与目标物主要基于疏水相互作用、尺寸排斥效应、*π-π*堆叠作用、氢键作用和范德华力,选择性有限。因此,未来需要开发出高亲水性、高选择性的新型COFs或者COFs复合材料,以满足不同应用场景下的需求。
